# Parkinsonian phenotype in Machado-Joseph disease (MJD/SCA3): a two-case report

**DOI:** 10.1186/1471-2377-11-131

**Published:** 2011-10-24

**Authors:** Conceição Bettencourt, Cristina Santos, Paula Coutinho, Patrizia Rizzu, João Vasconcelos, Teresa Kay, Teresa Cymbron, Mafalda Raposo, Peter Heutink, Manuela Lima

**Affiliations:** 1 Center of Research in Natural Resources (CIRN), University of the Azores, Ponta Delgada, Portugal; 2 Department of Biology, University of the Azores, Ponta Delgada, Portugal; 3 Institute for Molecular and Cell Biology (IBMC), University of Porto, Porto, Portugal; 4 Laboratorio de Biología Molecular, Instituto de Enfermedades Neurológicas, Fundación Socio-Sanitaria de Castilla-La Mancha, Guadalajara, Spain; 5 Unitat Antropologia Biològica, Dep. Biologia Animal, Biologia Vegetal i Ecologia, Universitat Autònoma de Barcelona, Bellaterra (Barcelona), Spain; 6 Department of Neurology, Hospital São Sebastião, Feira, Portugal; 7 Section of Medical Genomics, Department of Clinical Genetics, VU University Medical Center, Amsterdam, the Netherlands; 8 Department of Neurology, Hospital of Divino Espirito Santo, Ponta Delgada, Portugal; 9 Department of Clinical Genetics, Hospital of D. Estefania, Lisbon, Portugal

## Abstract

**Background:**

Machado-Joseph disease (MJD), or spinocerebellar ataxia type 3 (SCA3), is an autosomal dominant neurodegenerative disorder of late onset, which is caused by a CAG repeat expansion in the coding region of the *ATXN3 *gene. This disease presents clinical heterogeneity, which cannot be completely explained by the size of the repeat tract. MJD presents extrapyramidal motor signs, namely Parkinsonism, more frequently than the other subtypes of autosomal dominant cerebellar ataxias. Although Parkinsonism seems to segregate within MJD families, only a few MJD patients develop parkinsonian features and, therefore, the clinical and genetic aspects of these rare presentations remain poorly investigated. The main goal of this work was to describe two MJD patients displaying the parkinsonian triad (tremor, bradykinesia and rigidity), namely on what concerns genetic variation in Parkinson's disease (PD) associated loci (*PARK2, LRRK2, PINK1, DJ-1, SNCA, MAPT, APOE*, and mtDNA *tRNA*^*Gln *^T4336C).

**Case presentation:**

Patient 1 is a 40 year-old female (onset at 30 years of age), initially with a pure parkinsonian phenotype (similar to the phenotype previously reported for her mother). Patient 2 is a 38 year-old male (onset at 33 years of age), presenting an ataxic phenotype with parkinsonian features (not seen either in other affected siblings or in his father). Both patients presented an expanded *ATXN3 *allele with 72 CAG repeats. No PD mutations were found in the analyzed loci. However, allelic variants previously associated with PD were observed in *DJ-1 *and *APOE *genes, for both patients.

**Conclusions:**

The present report adds clinical and genetic information on this particular and rare MJD presentation, and raises the hypothesis that *DJ-1 *and *APOE *polymorphisms may confer susceptibility to the parkinsonian phenotype in MJD.

## Background

Autosomal dominant cerebellar ataxias (ADCA) constitute a heterogeneous group of neurodegenerative disorders, which involve, besides other systems, the extrapyramidal system in a highly variable manner. Within this group is Machado-Joseph disease (MJD; MIM #109150), also known as spinocerebellar ataxia type 3 (SCA3), that presents extrapyramidal motor signs (EPS) more frequently than the other subtypes of ADCA [1]. Even within MJD, the EPS may vary, sometimes with marked dystonic postures, others with isolated parkinsonian features or even, though rarely, with the parkinsonian triad (resting tremor, bradykinesia and rigidity) [2]. Although the causative mutation of MJD is known to be an expansion of a CAG repeat motif (consensually more than 52 repeats) in the coding region of the *ATXN3 *gene [3], the size of the of the repetitive tract does not completely explain the clinical heterogeneity observed in this disorder, namely concerning the presence of EPS.

There are only a few reports of a Parkinson's disease (PD)-like phenotype in molecularly confirmed MJD patients [4-6], and thus this particular MJD phenotype remains poorly documented, namely from the genetic point of view. The cases reported in the literature showed an initial phenotype indistinguishable from PD and a levodopa positive response. Only after several years of progression the characteristic features of MJD, namely cerebellar signs, were observed. The parkinsonian phenotype has been suggested to be more common in MJD patients of African descent, although rare in those of European origin [7].

MJD phenotypes with EPS, particularly those with a PD-like phenotype, may result from epistatic effects of variants in other loci. As primary candidates are PD associated loci, namely *PARK2, LRRK2, PINK1, DJ-1, SNCA, MAPT*, and *APOE *(e.g. [8-10]). Variation in mitochondrial DNA (mtDNA) has also been shown to have an impact on neurodegenerative diseases' phenotype, and the *tRNA*^*Gln *^T4336C mutation was previously associated with Alzheimer's disease (AD) and PD [11]. The main goal of this work was to describe two unrelated MJD patients displaying the parkinsonian triad, for which the genetic variation (mutations and/or polymorphisms) in PD associated loci was investigated.

## Case Presentation

Out of a series of 70 molecularly confirmed MJD patients from the Azores Islands (Portugal), from whom DNA samples were available (extracted from blood samples, which were collected after obtaining written informed consent), two patients presenting the parkinsonian triad, and belonging to distinct MJD families (one from São Miguel Island and other from Graciosa Island), were identified.

### Patient 1

**Figure 1:**
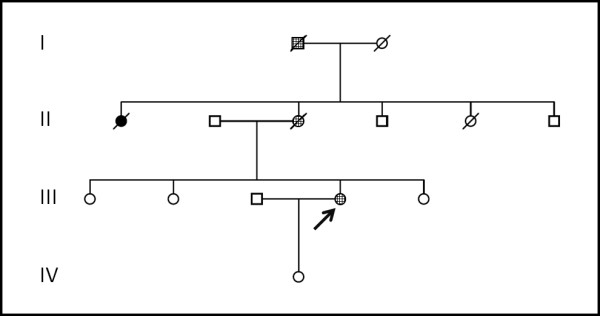
**Pedigree of Patient 1.** Black filled figures represent MJD patients; Crosshatched figures represent MJD patients with a parkinsonian phenotype.

Patient 1 is a 40 year-old female patient (Figure 1; III-4). At the age of 30 years (Table 1), she noticed slowness of movements. At examination, she had a poor mimic and marked bradykinesia. She responded well to L-dopa, which she maintained for 8 years. At this time she was hospitalized because of neurological aggravation and depression. She presented, besides a prominent parkinsonian syndrome with frequent on-off phenomena, cerebellar dysarthria and general incoordination, axial and upper limbs dystonia, upwards gaze limitation with upwards gaze nystagmus, generalized hyperreflexia and bilateral Babinski sign, as well as marked gait ataxia. Her mother (Figure 1; II-3) also had exhibited a parkinsonian phenotype. By the age of 34, she noticed difficulty in walking and in the fine movements of the hands. First examined at 36, she had bradykinesia, including amimia, bilateral hypertonia, without tremor, nor incoordination. The gait was purely parkinsonian, with small steps and no balance of the arms. She responded well to L-dopa, reaching a normal neurological examination under 300 mg + 75 mg of the inhibitor. Examined 12 years later, she was still under L-dopa treatment. Besides her parkinsonian syndrome and extreme diskynesias, she had a deep depression, a moderate gait and lower limbs' ataxia, cerebellar dysarthria, and marked upwards gaze limitation. The maternal grandfather of Patient 1 (Figure 1; I-1) had an onset at 63 years of age with slowly progressive gait ataxia, followed by dysarthria, diplopia and dysphagia. Examined the first time with 8 years of evolution, he had upwards gaze limitation and horizontal squint, moderate peroneal atrophy with generalized arreflexia, plantars in flexion, slight dysmetria in the upper limbs, marked dysmetria in the lower limbs and ataxic gait possible with unilateral support. Four years later mild Parkinson's signs were observed, with resting tremor in the upper limbs and bilateral rigidity. He died two years later aged 77 years.

**Table 1:** Characteristics of Patient 1 and Patient 2

Characteristics	Patient 1	Patient 2
**Age at Onset**	30	33
**Onset Mode**	Pure PD signs	PD plus cerebellar signs?
**Family history for PD features**	Mother and grandfather	NP
***ATXN3 *(CAG)n tract length**		
Normal Allele	14	29
Expanded Allele	72	72
***PARK2 *Gene Mutations**	NP	NP
***LRRK2 *Gene Mutations**	NP	NP
***PINK1 *Gene Mutations**	NP	NP
***DJ-1 *Gene**		
**Mutations**	NP	NP
**Polymorphisms**		
g.168_185del	Del/Del	Ins/Del
rs7517357	T/T	C/T
rs2641116	G/G	T/G
rs56327722	A/A	G/A
***SNCA *Gene**		
REP1	261/261	261/261
***MAPT *Gene**		
rs242557	A/G	G/G
Haplotype	H1/H2	H2/H2
***APOE *Gene**		
genotype	ε2/ε3	ε2/ε3
**mtDNA**		
T4336C mutation	NP	NP
Haplogroup	H	H

NP - Not present

### Patient 2

**Figure 2:**
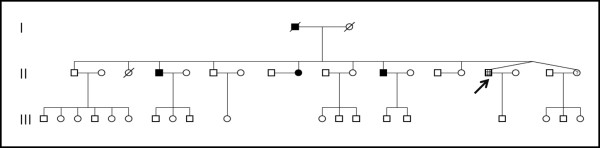
**Pedigree of Patient 2.** Black filled figures represent MJD patients; Crosshatched figures represent MJD patients with a parkinsonian phenotype.

Patient 2 is a 38 year-old male patient (Figure 2: II-16). He reported complaints of left hand's tremor since he was 33 years old. In neurological examination at the age of 35 years, he presented slight tremor of the left hand and of the L-lower limb. There was a cog-wheel rigidity of the left side, slight ataxia, hyperreflexia and right Babinski sign. The EPS responded consistently to L-dopa. His MRI was normal. His father (Figure 2: I-1) was only observed after 18 years of disease progression (reported onset at 42 years old), but did not present EPS. Furthermore, none of the remaining affected siblings (Figure 2: II-4, II-9, and II-12) present parkinsonian features.

### Molecular analysis

The *ATXN3 *(CAG)_n _tract size was determined as previously described [12]. All the exons and exon-introns boundaries of *PARK2, LRRK2, PINK1 *and *DJ-1 *genes were amplified by PCR. The resulting PCR products were purified by Exo-SAP treatment (GE Healthcare) and sequenced using the BigDye^® ^Terminator v3.1 Cycle Sequencing Kit (Applied Biosystems). Multiplex Ligation-Dependent Probe Amplification (MLPA) was used, according to Djarmati *et al*. [13], to search for large deletions or duplications in *PARK2, PINK1 *and *DJ-1*. The *SNCA *REP1 allele-length variants were genotyped as previously described [9]. In *MAPT *locus, a SNP (rs242557) was genotyped by a custom Taqman assay according to the manufacturer's specifications, and its genotype was used to infer *MAPT *haplotypes. The *APOE *genotypes were determined according to Bettencourt *et al*. [14]. The mtDNA haplogroup was established as previously described by Santos *et al*. [15], and the region encompassing the T4336C mutation was amplified by PCR, according to Ramos *et al*. [16]. PCR products were purified using the JETQUICK PCR Purification Spin Kit (Genomed) and sequenced, using the BigDye^® ^Terminator v3.1 Cycle Sequencing Kit (Applied Biosystems), according to the manufacturer's specifications.

Both patients presented 72 CAG repeats in their expanded *ATXN3 *allele (Table 1), which corresponds to the mode for the expanded alleles in the Azorean MJD series [17]. In *PARK2, LRRK2, PINK1, DJ-1 *and mtDNA *tRNA*^*Gln*^, no known pathogenic mutations were detected in the analyzed regions (Table 1). Several polymorphisms were found at the *DJ-1 *gene (Table 1). Results for the *SNCA *REP1, *MAPT *and *APOE *loci, as well as for the mtDNA haplogroup, are shown in Table 1.

Although MJD presents EPS more frequently than the other ADCAs, the manifestation of the parkinsonian triad is rare, being present in less than 3% of the analyzed MJD series. Even though the two studied cases are distinct, the grandfather-mother-daughter transmission in the family of Patient 1, and the heterogeneity presented by the family of Patient 2, suggests a supporting genetic background, e.g. modifier gene(s).

Mutations in the *PARK2 *gene have been described as the most frequent in the cases of early onset PD [18]. However, none of the studied patients presented mutations in this gene. Similarly, no mutations were identified in *LRRK2, PINK1 *and *DJ-1 *genes, or in position 4336 of mtDNA.

Given the fact that both patients belong to a typically European mtDNA haplogroup [15], the hypothesis of an African background, in which the parkinsonian phenotype seems more common [7], was not supported, at least in terms of maternal origin.

Association studies have shown that certain alleles of the *SNCA *REP1 locus, as well as the presence of two copies of *MAPT *H1, increase the risk of PD (e.g. [9,10]). In the patients analyzed, however, the homozygousity for the 261 allele (the commonest among Caucasians [9]), and the presence of at least one *MAPT *H2 was confirmed.

Although previous studies [19,20] failed to find an association between *DJ-1 *g.168_185del polymorphism and PD, De Marco *et al*. [21], using a larger series of PD patients, have reported that several *DJ-1 *polymorphisms, including those observed in the present study and reported in Table 1, are significantly associated with PD.

The *APOE *ε2 has been associated with increased risk of sporadic PD [8], and with an earlier onset of PD (e.g. [22]). The studied patients presented both the *APOE *ε2/ε3 genotype.

In non-Parkinsonian patients (N = 55) from the Azorean MJD series (data not shown), the *DJ-1 *g.168_185del was found in 23% of the alleles, not differing from the frequency of this deletion in other non-Parkinsonian Caucasian populations studied previously (e.g. [19-21]). The *APOE *ε2 allele was present in only 4% of the alleles, which corresponds to a lower frequency than the one previously reported for this allele in the general Azorean population [14]. Besides the two Parkinsonian MJD patients studied here, only 2 additional Azorean patients (non-Parkinsonian) carry both the *DJ-1 *g.168_185del and the *APOE *ε2 allele.

## Conclusions

The present report, in these two MJD patients, adds information on this rare phenotype, by allowing a deeper knowledge of the clinical and genetic variation present in such particular cases. Analysis of both pedigrees supports a genetic background influence. Although we cannot conclude on the presence of associations by studying only two Parkinsonian patients, the present results are in agreement with previous studies on Parkinson's disease. The implication of these findings, on what concerns a possible epistatic effect of the *DJ-1 *g.168_185del and the *APOE *ε2 allele on the MJD phenotype deserves to be tested, using an appropriate study design, namely on what concerns adequate sized parkinsonian MJD series (presently unavailable, to our knowledge).

## Consent

Written consent was obtained from both patients for the publication of this case report. A copy of written consent is available for review by the Editor-in-Chief of this journal.

## Competing interests

The authors declare that they have no competing interests.

## Authors' contributions

CB participated in the design of the study, carried out part of the molecular analysis and drafted the manuscript. CS, PR, TC, and MR participated in the molecular analysis. PC have collected part of the clinical data and contributed to draft the manuscript. TK and JV were involved in the sampling process and in the collection of the clinical data. PH participated in the design and discussion of the study. ML participated in the design and coordination of the study, and helped to draft the manuscript. All authors read and approved the final manuscript.

## Pre-publication history

The pre-publication history for this paper can be accessed here:

http://www.biomedcentral.com/1471-2377/11/131/prepub
